# Your fitness-sharing is a reminder for my poor body: how fitness posts exposure on social media harms female body esteem

**DOI:** 10.3389/fpsyg.2025.1515575

**Published:** 2025-03-19

**Authors:** Xiumei Yan, Jun Yan, Chuhan Tan, Yu Fu, Shuqing Wang

**Affiliations:** ^1^School of Education and Psychology, University of Jinan, Jinan, China; ^2^Qingdao Beiyang Architectural Design Co., LTD., Shandong, China

**Keywords:** online fitness, objectification theory, body surveillance, body esteem, social media, sport psychology

## Abstract

**Background:**

Prior literature suggests that fitness posts exposure on social media increases female body image concerns. However, little research has been conducted to examine the effect of fitness posts exposure on female body esteem.

**Objective:**

Given that, two studies were conducted to investigate how fitness posts exposure on social media exerted an influence on female body esteem.

**Method and results:**

By using a questionnaire survey (*n* = 270), we in Study 1 measured participants’ frequency of fitness posts exposure on social media, body esteem, body surveillance, and appearance contingent self-worth. The results showed that fitness posts exposure was significantly and positively related to female body esteem, and body surveillance played a mediating role between them. And this mediation was further moderated by appearance contingent self-worth. Simple slope analysis showed that fitness posts exposure had a significantly positive prediction on body surveillance at the high level of appearance contingent self-worth, but the prediction of fitness posts exposure was not significant at the low level of appearance contingent self-worth. Study 2 was a lab experiment, in which we randomly assigned 180 female undergraduates to the fitness-appearance exposure condition, the fitness-performance exposure condition, and the travel image exposure condition. Then, we asked participants to report body surveillance and body esteem. The results showed that participants in the fitness-appearance exposure condition reported higher body surveillance and lower body esteem than participants in the other two conditions. Consistent with Study 1, Study 2 revealed the mediating role of body surveillance between exposure condition and body esteem.

**Conclusion:**

Fitness posts exposure produces a negative influence on female body esteem via the mediation of body surveillance, and this pattern is more obvious among women with high appearance contingent self-worth. Appearance-relevant content involved in fitness posts, rather than pure fitness performance, harms female body esteem.

## 1 Introduction

When we are waiting for our meal in a restaurant or waiting for a bus at a bus station, we often involuntarily use our cellphones to seek possibly interesting information on social media platforms or idly browse status updates of online friends. Today, social media is popular around the world, which not only effectively enriches people’s leisure time, but also provides a novel approach to establishing and maintaining interpersonal relationships (Carr et al., 2016; Ellison et al., 2007; Godard and Holtzman, 2024). Prior literature suggests that social media has become a part of our lives and produced a widespread and profound effect on our physical health and psychological well-being (Krause et al., 2021; O’Day and Heimberg, 2021; Padín et al., 2021; Steers et al., 2014).

For many young females, body image is regarded as an important concern, even serving as a primary source of their self-worth (Perloff, 2014; Yang et al., 2020). Compared to older females and males, young females tend to pay more attention to the content relevant to fitness on various of social media platforms. And they are also pleased to share their exercise data, fitness selfies, or achievements on social media platforms (Raggatt et al., 2018; Roberts and Gettman, 2004). Past research suggests that while fitness-sharing on social media may possess positive intentions (e.g., promoting physical exercises and healthy eating), such fitness posts can motivate women to pursue perfect ideal body images (Donovan and Uhlmann, 2022; Prichard et al., 2018). However, because the so-called idealized body images on social media are hard to achieve, it is almost inevitable that fitness posts exposure exerts a detrimental effect on female body esteem.

Body esteem refers to “self-evaluations of one’s body or appearance,” which is considered to be an important domain of self-esteem, especially for young females (Mellor et al., 2010; Mendelson et al., 1996). It is somewhat surprising that, to date, little research has been conducted to examine the effect of fitness posts exposure on female body esteem while a large body of research suggests that fitness posts exposure can raise women’s body image concerns (e.g., Arroyo and Brunner, 2016; Prichard et al., 2020; Tiggemann and Zaccardo, 2015). To fill this gap, the present research was conducted to exclusively investigate whether and how fitness posts exposure on social media exerted an influence on female body esteem. By first examining the effect and mechanisms of fitness posts exposure — a specific social media activity — on female body esteem, the present research will contribute to revealing how social media affects women’s self-concept in a relatively focused way.

## 2 Literature review

### 2.1 Fitness posts on social media platforms

Social media generally refers to those online websites or mobile applications which allow users to autonomously create and share their favorite content, such as Facebook, Instagram, WeChat, TikTok and so on (Kaplan and Haenlein, 2010; Kaplan and Mazurek, 2018). Compared to traditional mass media, social media allows users to autonomously determine what they will view and publish on specific platforms, whether they will make the published content visible to all users, and whom they will communicate with (Ellison and Boyd, 2013). For young females, pursuing an ideal body image seems to be a common goal, which motivates them to sweat it out at the gym, learn about some fitness tips on social media platforms, and even suffer from disordered eating for a long time (Prichard and Tiggemann, 2008; Raggatt et al., 2018; Slater and Tiggemann, 2010). Thus, it is not surprising that young females often share some fitness-relevant tips, quotes, specific performance records, and their progress on social media platforms. In the present research, such online content relevant to fitness is collectively called fitness posts (Arroyo and Brunner, 2016).

Researchers have pointed out that there are diverse motivations behind the fitness posts sharing, and the majority of them actually hold positive goals, such as focusing on a healthy lifestyle or promoting physical health (Prichard et al., 2020; Raggatt et al., 2018). However, regardless of the motivations underlying fitness posts, empirical literature reveals that exposure to fitness posts increases body image concerns and leads to lower body self-evaluation or eating disorders (Arroyo and Brunner, 2016; Guo et al., 2022; Prichard et al., 2020; Tiggemann and Zaccardo, 2015). An important reason for this phenomenon is that, social media platforms often encourage female users to display ideal bodies in order to attract attention from other users and increase their engagement on these platforms (e.g., providing multiple kinds of filters or free photo-editing service; Chang et al., 2019). In addition, given that getting a feeling of superiority over others is an effective way of elevating self-esteem, many female users are also willing to present an attractive body shape on social media platforms — the so-called positive self-representation tendency (Bij de Vaate et al., 2018; Ozimek and Förster, 2021). As a result, those women who are frequently exposed to fitness posts may mistakenly perceive that they have an inferior body shape due to the failure of achieving ideal beauty standards, thus harming their body esteem.

### 2.2 Fitness posts exposure and female body esteem

Self-esteem is traditionally defined as a global positive or negative self-evaluation (Rosenberg, 1965). Similarly, body esteem refers to an overall evaluation of an individual toward his/her physical appearance, with high body esteem indicating positive self-evaluation toward physical appearance and low body esteem indicating negative self-evaluation toward physical appearance (Mendelson et al., 1996). In past research, although global self-esteem and body esteem sometimes are interchangeably to describe subjective feelings of oneself, they are considered to be different conceptual constructs (Mendelson et al., 2002). Body esteem is identified as an important predictor of global self-esteem, but what to extent body esteem will determine one’s global self-esteem depends on a variety of factors, such as age, gender, and even race (Henriques and Calhoun, 1999; Tyler et al., 2008). Additionally, body esteem is also different from body satisfaction — body satisfaction refers to the degree to which an individual is satisfied with his/her body weight/shape, while body esteem is characterized by feelings about how one looks or overall appearance (Tyler et al., 2008).

According to our previous reasoning, due to the unattainableness of ideal bodies on social media, it is very likely that fitness posts exposure raises a negative effect on female body esteem. Partially supporting this speculation, several empirical studies have demonstrated the close link between fitness posts exposure and lower body self-evaluation (Arroyo and Brunner, 2016; Hooper et al., 2024; Prichard et al., 2020; Tiggemann and Zaccardo, 2015). For example, by asking 488 undergraduates to complete an online survey, Arroyo and Brunner (2016) explored whether fitness posts exposure on social networking sites would significantly produce a prediction on negative body talk. The results showed that, for both male and female undergraduates, higher fitness posts exposure was significantly related to more negative body talk, manifested by more negative self-evaluations of their bodies and lower body satisfaction (Arroyo and Brunner, 2016). In another study, researchers employed a lab experiment in which 130 female undergraduates were presented with fitness images (fitness exposure condition) or travel images (control condition) on iPad, and found that female undergraduates in the fitness exposure condition reported more negative mood and body dissatisfaction, and lower state appearance self-esteem than female undergraduates in the control condition (Tiggemann and Zaccardo, 2015). Similarly, Prichard et al. (2020) also found that young females exposed to fitspiration images showed higher negative mood and body dissatisfaction compared to those exposed to travel images. Taken together, while the existing research indicates the feasibility that fitness posts exposure can produce a detrimental effect on female body esteem, little research has been conducted to provide direct evidence for the causality between them.

### 2.3 The mediating role of body surveillance

In the current research, the objectification theory was employed to provide a theoretical framework to elucidate the underlying mechanisms between fitness posts exposure and female body esteem (Fredrickson and Roberts, 1997). The objectification theory contends that, in Western society, women are continuously inculcated with the belief that their physical appearance is a primary source of their value and worth, even over their body function and competence (Fredrickson and Roberts, 1997; Loughnan and Vaes, 2017; Roberts et al., 2017). Past research suggests that viewing appearance-relevant content on social media contributes to the self-objectification of women and leads to higher body surveillance of them (Morry and Staska, 2001; Tylka et al., 2023; Tylka and Sabik, 2010; Vandenbosch et al., 2022). For example, Morry and Staska (2001) found that those female undergraduates exposed to ideal bodies presented in magazines were more likely to report higher self-objectification tendencies. In another study, researchers found that undergraduates who perceived more appearance feedback from others (e.g., *someone made some facial expression when looking at their body*) often displayed higher body surveillance, a typically behavioral manifestation of female self-objectification (Tylka and Sabik, 2010).

With respect to fitness posts on social media, as these fitness posts often highlight appearance-relevant benefits, we reason that exposure to fitness posts will increase women’s body surveillance. Supporting our reasoning, Donovan and Uhlmann (2022) recently found that young females with greater fit ideal internalization tended to show higher body surveillance. Not too long ago, Seekis et al. (2020) conducted an online questionnaire survey among female undergraduates to explore the relationship and the underlying mechanisms between specific activities on SNS and body concerns (Seekis et al., 2020). They found that although exposure to fitness-related content did not significantly predict body surveillance, viewing celebrity or fashion content — some typically appearance-relevant activities — significantly and positively predicted body surveillance of female undergraduates. Based on such findings, we reason that fitness posts exposure on social media will have a significant and positive prediction on body surveillance of women.

Considering that ideal body images on social media are hard to attain in reality, frequent body surveillance of female users should lead to lower self-evaluation of their bodies. For instance, Aubrey (2007) found that exposure to specific objectifying content on mass media (e.g., thin-ideal bodies) empowered females to engage in high body surveillance, and high body surveillance was further related to obvious body shame and appearance anxiety. In an online experiment, researchers randomly assigned participants to the appearance group (asking participants to write about their appearance) or the personality group (asking participants to write about their personality), and then measured their body surveillance, body shame, and other variables (Moya-Garófano and Moya, 2019). The results showed that instructing participants to focus on their own appearance induced higher body surveillance of female participants, and higher body surveillance induced greater body shame of them. Following the above reasoning, under the framework of the objectification theory, we hypothesize that more fitness posts exposure on social media leads to higher body surveillance, which further harms their body esteem.

### 2.4 The moderating role of appearance contingent self-worth

Contingent self-worth means a premise of achieving a feeling of self-worth is that an individual must meet some external standards (e.g., appearance or academic performance; Crocker and Wolfe, 2001; Deci and Ryan, 1995). In nature, contingent self-worth represents a conditional self-acceptance. Correspondingly, appearance contingent self-worth refers to the degree to which an individual determines their worth and value based on their physical appearance (Noser and Zeigler-Hill, 2014; Sanchez and Crocker, 2005; Sharma et al., 2024). Those individuals who base their self-worth on physical appearance may attempt to achieve external standards of beauty, even if such standards are unattainable. As a consequence, individuals with high appearance contingent self-worth tend to display frequent body surveillance (Overstreet and Quinn, 2012; Stapleton et al., 2017). In contrast, individuals with low appearance-contingent self-worth are believed to exhibit fewer body image concerns and body surveillance (Modica, 2019).

In the present research, we hypothesize that fitness posts exposure is more likely to have a significant prediction on body surveillance for women with high appearance contingent self-worth than for those with low appearance contingent self-worth. Namely, appearance contingent self-worth may moderate the relationship between fitness posts exposure and body surveillance. Several studies have provided support for this hypothesis (Adams et al., 2017; Modica, 2019; Noser and Zeigler-Hill, 2014; Overstreet and Quinn, 2012). For example, when exploring the relationships between specific Facebook activities and body esteem in adult women, Modica (2019) found that there was a significantly positive correlation between appearance contingent self-worth and body surveillance. Overstreet and Quinn (2012) also found that investing self-worth in appearance was associated with frequent body surveillance. In addition, Bardone-Cone et al. (2017) found that appearance contingent self-worth moderated the relationship between perfectionism and disordered eating, a common downstream effect related to female self-objectification. Specifically, there was a stronger association between perfectionism and disordered eating when appearance contingent self-worth was high than when appearance contingent self-worth was low. Taken together, the existing research consistently demonstrates that women with high appearance contingent self-worth tend to show high body surveillance.

### 2.5 Specific types of fitness posts and female body esteem

In addition to the above major concerns, the present research also carries an explorative purpose — exploring whether specific types of fitness posts will exert different influences on female body esteem. While the majority of research reveals a detrimental effect of fitness posts exposure on body image, a small number of research does not find a significant relationship between fitness posts exposure and body image (McComb and Mills, 2022; Prichard et al., 2020; Seekis et al., 2020; Tiggemann and Zaccardo, 2015). We speculate that such mixed findings may be explained by specific types of fitness posts. For instance, by randomly asking 106 female undergraduates to view one of three sets of images (thin ideal, athletic ideal, or muscular ideal) followed by a bout of exercise, Robinson et al. (2017) found that acute exposure to athletic ideal and thin ideal images contributed to body dissatisfaction, but exposure to muscular ideal images did not show this pattern. This finding suggests that it may be necessary to identify the effect of specific types of fitness posts on female body esteem. Following our previous reasoning, we postulate that appearance-relevant content involved in fitness posts, rather than “pure” fitness, will have a detrimental effect on female body esteem. Given that, we divided fitness posts into two subtypes — fitness posts containing appearance-relevant content and fitness posts containing pure fitness records, then examined whether such fitness posts would have different effects on body esteem. We reason that fitness posts containing appearance-relevant content, rather than fitness posts only containing fitness records, will produce a negative effect on body esteem.

## 3 The present research

The primary goal of the present research was to examine whether and how fitness posts exposure would exert an influence on female body esteem. Additionally, we also examined whether specific types of fitness posts would have different influences on body esteem. According to previous reasoning, we proposed four specific hypotheses.

*H1:* Fitness posts exposure exerts a negative influence on female body esteem.

*H2:* Fitness posts exposure produces a detrimental effect on female body esteem via the mediating role of body surveillance.

*H3:* Fitness posts exposure is more likely to contribute to high body surveillance for women with high appearance contingent self-worth than those with low appearance contingent self-worth, resulting in the moderating role of body surveillance between fitness posts exposure and body esteem.

*H4:* Fitness posts containing appearance-relevant content, rather than fitness posts only containing fitness records, produces a negative effect on female body esteem.

To test the above hypotheses, two studies were conducted. Study 1 was a questionnaire survey, in which we examined the relationship between fitness posts exposure and female body esteem, the mediating role of body surveillance between them, and the moderating role of appearance-contingent self-worth. Study 2 was a lab experiment, in which we randomly exposed participants to three types of images (fitness images containing appearance-relevant content, fitness images only containing fitness records, and travel images). Then, we examined whether there were any differences in body esteem across the three conditions and the mediating role of body surveillance between fitness posts exposure and body esteem. In the present research, the sample of Study 2 was independent of that of Study 1.

## 4 Study 1

Study 1 was a correlational design, in which we employed the questionnaire survey to examine the relationship between fitness posts exposure and body esteem, and the possible mediating role of body surveillance between them. By doing so, we sought to provide initial support for Hypothesis 1 and 2. We also examined the possible moderating role of appearance-contingent self-worth for the mediation of body surveillance, which would provide support for Hypothesis 3.

### 4.1 Method

#### 4.1.1 Participants

The sample size was determined prior to conducting any data analyses. Based on the calculation of G*power 3.1 (Faul et al., 2009), a presupposed *β* = 0.2 in a linear regression equation requested at least 262 participants. Considering possible invalid data, we finally recruited 270 female undergraduates to take part in Study 1. A total of 11 participants were identified as invalid participants due to the following reasons: 4 for obviously abnormal responses (e.g., indicating 2 for all items in a scale and 3 for all items in another scale, or providing their answer in a circular order from 1 to 7), 5 for excessive omissions, and 2 for indicating the same answer for all items. As a consequence, there were 259 participants involved in the final data analysis. All participants received 5 RMB for their participation. We reported detailed demographic information of participants in the Results section.

#### 4.1.2 Materials

##### 4.1.2.1 Fitness posts exposure

We assessed participants’ exposure frequency to fitness posts on social media via the scale developed by Arroyo and Brunner (2016). The scale consists of 6 items and these items include fitness picture, fitness performance, fitness inspiration, and so on. For instance, an example item presented here was “*on social media, how often will you access pictures of others working out or at the gym*.” For each item, participants needed to indicate their agreement on the 7-point scale (1 = *never*, 7 = *always*). Participants’ exposure frequency of fitness posts on social media was calculated by summing the score on each item, with higher scores indicating higher exposure frequency to fitness posts. The scale has been used in Chinese culture and demonstrates good suitability (Yang et al., 2023). In Study 1, the internal consistency coefficient of the scale was 0.91.

##### 4.1.2.2 Body surveillance

Following previous research (Yang et al., 2023), the Chinese version of the Body Surveillance subscale of the Objectified Body Consciousness scale developed by McKinley and Hyde (1996) was applied to assess the tendency to monitor one’s appearance. The Body Surveillance subscale includes 8 items in total (e.g., *during the day, I think about how I look many times*) and participants needed to provide their agreement for each item on the 7-point scale (1 = *strongly disagree*, 7 = *strongly agree*). For each participant, after the item 1, 2, 3, 4, 7 and 8 were reversely scored, the score of body surveillance was calculated by averaging her scores on all items, with higher scores indicating greater body surveillance. Prior literature has demonstrated that the Body Surveillance subscale displays acceptable internal consistency among young adult women (Modica, 2019; Tylka and Wood-Barcalow, 2015). In Study 1, the internal consistency coefficient of the subscale was 0.90.

##### 4.1.2.3 Body esteem

Following previous research (Yang et al., 2020), the Body Esteem Scale for Adolescents and Adults (BESAA; Mendelson et al., 2001) was applied to measure individual’ body esteem in Study 1. The BESAA includes three subscales: Weight, Appearance, and Attribution, which can detect individuals’ “attitudes and feelings about their bodies and appearance” (Mendelson et al., 2001). Of three subscales, the 8-item Weight subscale detects one’s feelings and attitudes toward his/her weight (e.g., *I really like what I weigh*) and the 10-item Appearance subscale detects one’s feelings and attitudes toward his/her overall appearance (e.g., *I like what I look like in pictures*). The 5-item Attribution subscale reflects how one person thinks others evaluated his/her appearance (e.g., *other people consider me good looking*). For each item, participants needed to provide their agreement on the 5-point scale (1 = *strongly disagree*, 5 = *strongly agree*).

Due to lacking an available Chinese version of the BESAA, we first translated the English version of the BESAA into Chinese version following the back-translation procedure before carrying out the questionnaire survey (Brislin, 1980). Specifically, we invited two independent psychology Ph.D. students who were both fluent in English. One first translated the English version of the BESAA into Chinese, then the Chinese version was translated into English by the other one. Any discrepancies between the two English versions were further adjusted. Following the recommendation by Modica (2019), while the entire BESAA was administered to participants, only the Weight and Appearance subscales were calculated to represent body esteem. After deleting four items (items 1, 3, 9, and 11) with factor loadings lower than 0.45, a confirmatory factor analysis (CFA) performed on the Mplus 8.0 showed an acceptable model fit, *χ*^2^/df = 2.58, *p* < 0.001, CFI = 0.94, TLI = 0.92, RMSEA = 0.08, SRMR = 0.06. Following Modica (2019), all items in the modified Weight and Appearance subscales were summed to generate an overall score, with higher scores indicating higher body esteem. In Study 1, the internal consistency coefficient of the composite scale was 0.89.

##### 4.1.2.4 Appearance contingent self-worth

Following previous research (Modica, 2019), the Appearance subscale of the Contingencies of Self-Worth Scale (CSWS; Crocker et al., 2003) was used to measure the appearance contingent self-worth of participants. The subscale contained five items (e.g., *my self-esteem is influenced by how attractive I think my face or facial features are*), and participants gave their agreement for each item on the 7-point scale (1 = *strongly disagree*, 7 = *strongly agree*). For each participant, we calculated an appearance-contingent score by averaging the scores on all the items, with higher scores indicating higher appearance-contingent self-worth.

Due to lacking the Chinese version of the appearance contingent self-worth subscale, we developed the Chinese version of the subscale. Again, the back-translation procedure was applied to confirm the equivalence between the Chinese and English versions. The CFA was performed on Mplus 8.0 to confirm the construct of the appearance contingent self-worth subscale. After deleting the item 5 with low factor loading (0.33), the model showed a good fit index, *χ*^2^/df = 1.29, *p* = 0.28, CFI = 0.99, TLI = 0.99, RMSEA = 0.03, SRMR = 0.01. As a result, a total of 4 items were included in final data analysis. The internal consistency coefficient of the modified scale was 0.89.

##### 4.1.2.5 Global self-esteem

Prior research suggests that there is a close connection between body self-esteem and global self-esteem (e.g., Mendelson et al., 1996). So, it is possible that participants’ body self-esteem may fluctuate with trait global self-esteem. To control for this possible confounding factor, we used the Chinese version of the Rosenberg Self-Esteem Scale to measure participants’ trait global self-esteem (Rosenberg, 1965; Ji and Yu, 1999). The scale consisted of 10 items (e.g., *on the whole, I am satisfied with myself*) and participants needed to indicate their agreement for each item on the 4-point scale (1 = *strongly disagree,* 4 = *strongly agree*). After several items were reversely scored, we calculated the global self-esteem score by summing scores on all the items, with higher scores indicating higher global self-esteem. The Chinese version of the Rosenberg Self-Esteem Scale has been widely used in Chinese culture and displays a good suitability. In the current research, the internal consistency of the scale was 0.86.

##### 4.1.2.6 Demographic information

In addition to the above variables, we also collected some demographic information of participants, including their age, height, weight, nationality, residence (country/city), and social status. One thing we wanted to explain was that considering most college students were not clear about their family incomes, we did not measure participants’ objective social status. Rather, we assessed their subjective social status by asking them to mark an “X” next to one of 10 rungs on a ladder to indicate their family status within the whole Chinese society (Kraus et al., 2009).

#### 4.1.3 Procedure

The survey was carried out with a group of 40 ~ 60 participants. Prior to the formal survey, we told participants that we were interested in “undergraduates’ opinions and attitudes toward body shaping.” We also explicitly declared that they can determine their participation on a voluntary basis. If they were willing to continue the survey, they needed to assign the informed consent. After that, they successively complete the measures of fitness posts exposure, body surveillance, body esteem, global self-esteem, and appearance contingent self-worth. At the end of the questionnaire, participants reported their demographic information. When they completed the whole questionnaire, they were thanked, thoroughly debriefed, and paid. The survey lasted for approximately 15 min.

### 4.2 Results

#### 4.2.1 Descriptive statistical results

Most of the participants involved in Study 1 were Han nationality (268 Han, 1 Hui, and 1 Miao), whose mean age was 20.15 years old (*SD* = 0.76), ranging from 18.33 to 20.33 years old. One hundred and fifteen participants reported that they lived in the country (approximately 44.4%), and one hundred and forty-fourth participants reported that they lived in the city (approximately 55.6%). Body mass index (BMI) was calculated by dividing participants’ current weight (kg) by their height (m^2^). Participants’ average BMI was 19.94 (*SD* = 2.51), with a range of 13.89 ~ 28.12.

Correlations among variables were presented in Table 1, which showed that there were significant correlations among key variables. Specifically, fitness posts exposure was significantly and positively correlated with body surveillance, *r* = 0.34, *p* < 0.01, but negatively correlated with body esteem, *r* = −0.23, *p* < 0.01. Body surveillance was significantly and negatively correlated with body esteem, *r* = −0.44, *p* < 0.01. Appearance contingent self-worth was significantly and positively correlated with fitness posts exposure and body surveillance, *r*s = 0.26, 0.55, respectively, *p*s < 0.01. In contrast, appearance contingent self-worth was significantly and negatively correlated with body esteem and global esteem, *r*s = −0.41, −0.25, *p*s < 0.01.

**Table 1:** Means, standard deviations, and correlations between variables in Study 1.

	*M*	*SD*	Fitness posts exposure	Body surveillance	Body esteem	Appearance contingent self-worth	Global self-esteem	BMI	Age	Subjective social status
Fitness posts exposure	3.63	1.53	1							
Body surveillance	3.91	1.24	0.34^**^	1						
Body esteem	46.78	10.86	−0.23^**^	−0.44^**^	1					
Appearance contingent self-worth	3.33	1.46	0.26^**^	0.55^**^	−0.41^**^	1				
Global self-esteem	30.50	5.54	0.16^**^	−0.12	0.35^**^	−0.25^**^	1			
BMI	21.00	4.17	0.03	0.08	−0.11	0.03	0.08	1		
Age	20.15	0.76	0.11	0.02	0.04	0.11	0.10	0.21^**^	1	
Subjective social status	4.58	1.34	0.06	0.04	0.14^*^	0.03	0.18^**^	−0.04	−0.02	1

M, mean value; SD, standard deviation; BMI, body mass index. ^*^p < 0.05, ^**^p < 0.01.

Additionally, global self-esteem was significantly and positively correlated with fitness posts exposure and body esteem, *p*s < 0.01. Subjective social status was significantly and positively correlated with body esteem and global self-esteem, *p*s < 0.05.

#### 4.2.2 The mediating role of body surveillance

To test whether there was a significantly negative relationship between fitness posts exposure and body esteem, we conducted a linear regression equation in which body esteem was regressed on fitness posts exposure. Global self-esteem, age, subjective social status, residence (0 = country, 1 = city), and BMI were included in the equation as covariates. Before entering the equation, all variables were standardized. The results showed that fitness posts exposure had a significantly negative prediction on body esteem, *β* = −0.30, *t* = −5.31, *p* < 0.001, thus supporting Hypothesis 1.

To test whether body surveillance mediated the relationship between fitness posts exposure and body esteem, the Macro PROCESS (Model 4) developed by Hayes (2013) was used to examine this possible mediation^1^. By applying a resampling strategy (resampling 5,000 times in this study), the Macro PROCESS generates a 95% confidence interval around the indirect effect. If the interval does not contain a zero, the indirect effect will be considered to be reliable (Preacher and Hayes, 2008). The results showed that the overall model was significant, *R*^2^ = 0.34, *F*(7,251) = 18.36, *p* < 0.001. The mediating role of body surveillance between fitness posts exposure and body esteem was significant, *f* = −0.11, 95% CI [−0.18, −0.07]. The indirect effect approximately accounted for 38% of the total effect. Specific path coefficients showed that fitness posts exposure had a significantly positive prediction on body surveillance, *β* = 0.34, *t* = 5.80, *p* < 0.001, and body surveillance had a significantly negative prediction on body esteem, *β* = −0.33, *t* = −5.80, *p* < 0.001. When the mediating effect of body surveillance was taken into account, the coefficient between fitness posts exposure and body esteem was reduced from *β* = −0.30 (*p* < 0.001) to *β* = −0.19 (*p* < 0.01). The above analyses demonstrated the mediating role of body surveillance between fitness posts exposure and body esteem, thus supporting Hypothesis 2.

#### 4.2.3 The moderating role of appearance contingent self-worth

To examine whether the mediation of body surveillance between fitness posts exposure and body esteem would be moderated by appearance contingent self-worth, we again applied the Macro PROCESS (Model 7) developed by Hayes (2013) to examine this possible moderated mediation. The results revealed a significant moderated-mediation model, *R*^2^ = 0.37, *F*(8,250) = 18.56, *p* < 0.001. As shown in Table 2, the indirect effect of body surveillance was significant at both medium and high levels of appearance contingent self-worth, 95%CI [−0.12, −0.03], [−0.18, −0.06], but was not significant at the low level of appearance contingent self-worth, 95%CI [−0.09, 0.02].

**Table 2:** Indirect effects of body surveillance across high and low levels of appearance contingent self-worth.

Appearance contingent self-worth	Conditional indirect effect	*SE*	Boot LLCI	Boot ULCI
*M*−1*SD*	−0.03	0.03	−0.09	0.02
*M*	−0.07	0.03	−0.12	−0.03
*M* + 1*SD*	−0.11	0.03	−0.18	−0.06

M, mean value; SD, standard deviation; SE, standard error; LLCI, lower limit of confidence interval; ULCI, upper limit of confidence interval.

**Figure 1:**
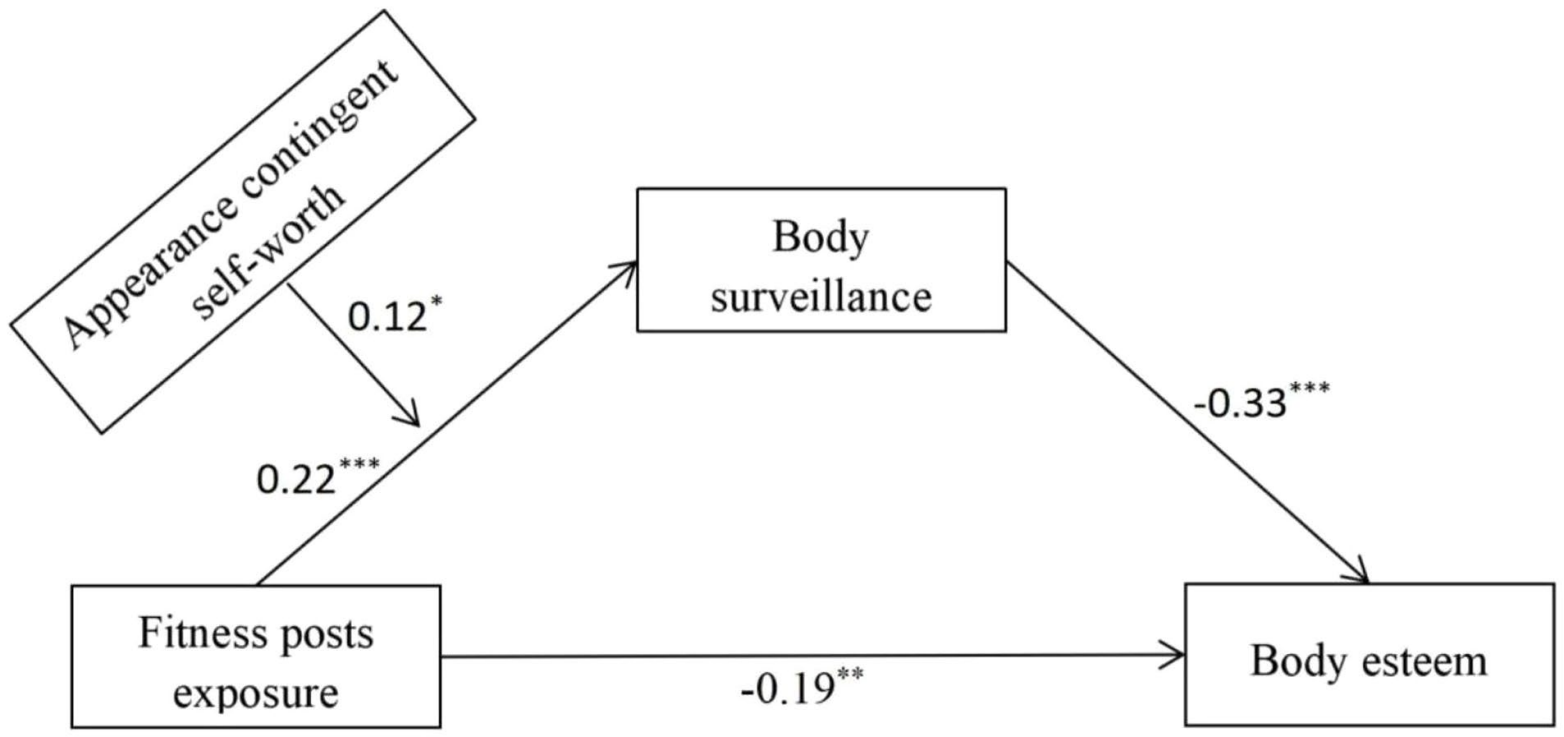
The mediating role of body surveillance between fitness posts exposure and female body esteem, and the moderating effect of appearance contingent self-worth for this mediation in Study 1. ^***^p < 0.001, ^**^p < 0.01, ^*^p < 0.05.

**Figure 2:**
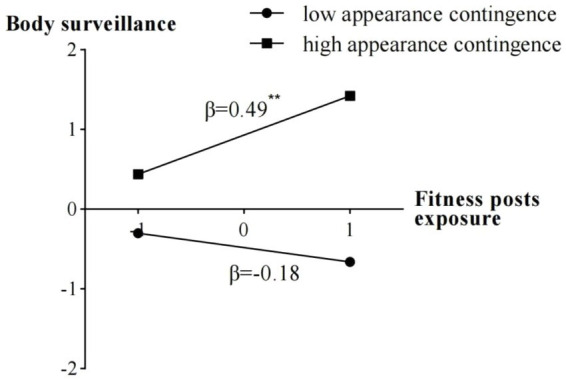
The prediction of fitness posts exposure on body surveillance across low and high appearance contingent self-worth conditions in Study 1. ^**^p < 0.01.

Specific path coefficients among variables were presented in Figure 1. As shown in the figure, fitness posts exposure had a significantly positive prediction on body surveillance, *β* = 0.22, *p* < 0.001, while body surveillance had a significantly negative prediction on body esteem, *β* = −0.33, *p* < 0.001. Importantly, appearance contingent self-worth showed a significant moderation for the relationship between fitness posts exposure and body surveillance, *β* = 0.12, *p* < 0.05. Figure 2 displayed the results of the simple slope analysis, which showed that fitness posts exposure had a significantly positive prediction on body surveillance at the high level of appearance contingent self-worth (the segment with black boxes), *β* = 0.49, *p* = 0.001, but fitness posts exposure did not have a significant prediction on body surveillance at the low level of appearance contingent self-worth (the segment with black dots), *β* = −0.18, *p* = 0.39. The above findings provided support for Hypothesis 3.

### 4.3 Discussion

In Study 1, we employed a questionnaire survey to investigate the relationship between fitness posts exposure and female body self-esteem, and the mediating mechanism between them. The results provided preliminary support for Hypothesis 1 and 2. That is, fitness posts exposure was significantly and negatively related to female body self-esteem, and body surveillance played a mediating role between them. Moreover, the mediating role of body surveillance was further moderated by appearance contingent self-worth.

Prior literature suggests that although exposure to fitness posts can promote the intention of pursuing physical exercise and a healthy lifestyle, it is also inevitable to cause excessive body image concerns (Raggatt et al., 2018; Tiggemann and Zaccardo, 2015). As a result, those women frequently exposed to fitness posts are more likely to invest a great deal of time and effort into body surveillance and body shaping. Unfortunately, as those so-called idealized bodies on social media actually are hard to achieve, most women frequently exposed to fitness posts tend to display lower self-evaluation. By firstly demonstrating the negative connection between fitness posts exposure and female body esteem, the present research adds new evidence for the potential detrimental-effect of fitness posts exposure on female self-concept. And the disclosure of the moderation of appearance contingent self-worth further clarified the condition under which fitness posts exposure may be more likely to exert a negative influence on women. That is, compared to women with low appearance contingent self-worth, those women with high appearance contingent self-worth are more vulnerable to fitness posts exposure due to frequent body surveillance.

There are two limitations existing in Study 1. Firstly, when we reasoned that fitness posts exposure on social media would harm female body esteem, a premise was that most fitness posts on social media highlighted appearance-relevant benefits. However, in Study 1, we assessed participants’ exposure frequency of fitness posts at a general level, and did not differentiate specific types of fitness posts. So, it is still unclear for us whether the effect of fitness posts exposure on body esteem will vary with specific types of fitness posts, thus failing to give a response to Hypothesis 4. Additionally, Study 1 in nature was a correlational design, which did not allow us to draw causal inferences among variables. Therefore, although our findings provided preliminary support for Hypothesis 1 that higher fitness posts exposure would lead to lower female body self-esteem, an alternative explanation in the opposite direction was also plausible. Namely, those women with lower trait body esteem paid more attention to fitness content on social media, and were also more likely to continually monitor their bodies per day. Given the above considerations, we conducted Study 2, in which we assigned participants to the three exposure conditions in the lab — fitness images exposure containing appearance-relevant content, fitness images exposure only containing performance records, and travel images exposure. Then, we investigated whether different exposure conditions would produce different effects on female body esteem. By doing this, we can draw causal inferences among key variables.

## 5 Study 2

We sought to achieve two major goals in Study 2. The first goal, as we have mentioned above, was to provide causal evidence for the effect of fitness posts exposure on female body esteem via conducting a lab experiment (Hypothesis 1). The second goal was to provide evidence for Hypothesis 4 by investigating whether the effect of fitness posts exposure on female body esteem would vary with specific types of fitness posts. In addition, we also examined whether the exposure condition would produce a detrimental effect on female body esteem via the mediating role of body surveillance, thus providing further evidence for Hypothesis 2.

### 5.1 Method

#### 5.1.1 Participants and design

The key design of Study 2 was a one-way analysis of variance (ANOVA) with the exposure condition as a three-level independent variable (fitness images containing appearance-relevant content, fitness images only containing performance records, and travel images). According to the calculation of G*power 3.1 (Faul et al., 2009), a presupposed medium effect size (*f* = 0.3) and a 0.05 significant level approximately required 177 participants in total. Based on this, we finally recruited 180 female undergraduates to participate in Study 2. All participants voluntarily took part in this study and signed the informed consent prior to the formal experiment. As return, they would receive 10 RMB (approximately 1.4 USD) after completing all tasks.

#### 5.1.2 Materials

##### 5.1.2.1 Exposure condition manipulation

**Figure 3:**
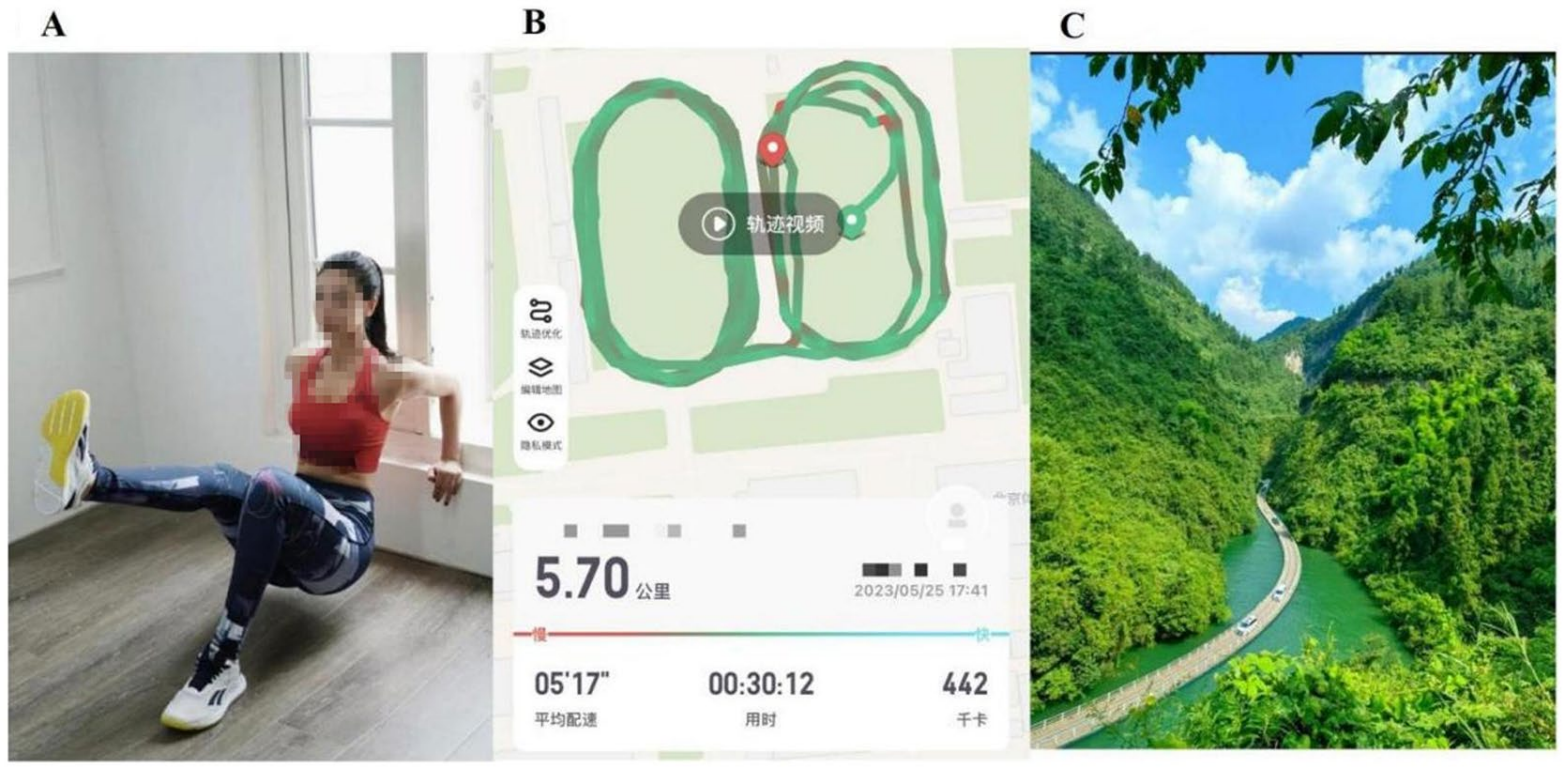
Example images in the three conditions of Study 2. Panel **(A)** was an example image in the fitness-appearance exposure condition. Panel **(B)** was an example image in the fitness-performance exposure condition. Panel **(C)** was an example image in the travel image exposure condition.

The exposure condition manipulation was alleged as a memory task. In this so-called memory task, participants were randomly assigned to one of the three conditions — the fitness-appearance exposure condition, the fitness-performance exposure condition, and the travel-image exposure condition. The fitness-appearance exposure condition consisted of 18 fitness images and 4 travel images, and importantly, each fitness image in the fitness-appearance exposure condition displayed a woman who was engaging in a specific fitness activity. The fitness-performance exposure condition consisted of 18 screenshots of running performance and 4 travel images. Those screenshots of running performance did not include any appearance-relevant content (e.g., selfie), and user information generated by fitness applications was pixelated. The travel image exposure condition consisted of 22 travel images and these images only captured some natural and cultural landscapes. All images in the fitness-appearance exposure and travel image exposure conditions were taken from publicly available sources on the Internet. The 18 images of running performances were voluntarily provided by some members of a WeChat group chat for fitness enthusiasts. Example images were presented in Figure 3. In a pretest, we invited 26 undergraduates to rate the perceived image quality in each condition, and did not find significant differences among the three conditions, *F*(2,50) = 0.03, *p* = 0.97.

In each condition, images were presented on the computer in a random order via the E-prime 3.0. Each image would stay on the screen for 10 s, then disappeared. After a blank screen for 800 milliseconds, the next image would appear on the screen. To increase participants’ involvement in the task, we explicitly reminded participants that they should pay attention to the details of images, and at the end of the whole task, they needed to answer several questions relevant to these images. Following Tiggemann and Zaccardo (2015), the effectiveness of the exposure manipulation was checked by asking participants to report their desires for fitness and travel in the future on the 7-point scale (1 = *never*, 7 = *strongly*).

##### 5.1.2.2 Body surveillance

As in Study 1, the Chinese version of the Body Surveillance subscale of the Objectified Body Consciousness scale developed by McKinley and Hyde (1996) was used to measure the body surveillance of participants. For each item of the Body Surveillance subscale, participants provided their agreement on the 7-point scale (1 = *strongly disagree*, 7 = *strongly agree*). After several items were reversely scored, body surveillance score was generated by averaging scores on all items, with higher scores indicating greater body surveillance.

##### 5.1.2.3 Body and global self-esteem

As in Study 1, the Rosenberg Self-Esteem Scale by Rosenberg (1965) was used to measure participants’ trait global self-esteem, and the modified Appearance and Weight subscales of the BESAA by Mendelson et al. (2001) were used to measure participants’ state body esteem. In line with Modica (2019), all items in the Weight and Appearance subscales were summed to generate a total score, with higher scores indicating higher body esteem. In Study 2, the internal consistency coefficient of the Rosenberg Self-Esteem Scale was 0.89, and the internal consistency coefficient of the BESAA was 0.91.

##### 5.1.2.4 Demographic information

In Study 2, the following demographic information was collected: age, height, weight, nationality, and subjective social status.

#### 5.1.3 Procedure

At the appointed time, participants arrived at the lab in group of 8 ~ 10. Then, a research assistant told them that they would complete a memory task and a self-evaluation task. They needed to assign the informed consent if they were willing to participate in this experiment. Next, they successively completed the so-called memory task (exposure condition manipulation), the Body Surveillance subscale, the modified BESAA, and the Rosenberg Self-Esteem Scale. Finally, they reported demographic information and completed the manipulation effectiveness check. Specifically, on the 7-point scale (1 = *not at all*, 7 = *very much*), they separately reported to what extent they intended to engage in fitness activities and go on a journey in the future. When participants had completed all tasks, they were thoroughly debriefed, thanked, and paid. The whole experiment lasted for approximately 20 min.

### 5.2 Results

#### 5.2.1 Manipulation check

According to the reasoning by Tiggemann and Zaccardo (2015), if exposure condition manipulation was successful, participants in the fitness-appearance and fitness-performance conditions should display greater fitness motivation than participants in the travel image exposure condition; correspondingly, participants in the travel image exposure condition should display greater travel motivation than participants in the other two conditions. Independent-samples *t* tests showed that participants in the both fitness-appearance and fitness-performance conditions reported greater fitness motivation than participants in the travel image exposure condition (*M*_appearance_ = 4.72, *M*_performance_ = 4.50, and *M*_travel_ = 4.00), *t*(118)_appearance-travel_ = 3.29, *p* = 0.001, *t*(116)_performance-travel_ = 2.47, *p* = 0.02. In contrast, participants reported greater travel motivation in the travel image exposure condition than participants in the other two conditions (*M*_appearance_ = 4.00, *M*_performance_ = 4.27, and *M*_travel_ = 4.83), *t*(116)_performance-travel_ = −3.97, *p* < 0.001, *t*(118)_appearance-travel_ = −2.75, *p* = 0.007. There were no significant differences in fitness or travel motivation between the fitness-appearance and fitness-performance conditions, *t*(116) = 0.93, *p* = 0.35, *t*(116) = −1.35, *p* = 0.18, respectively. The above results demonstrated the effectiveness of exposure condition manipulation.

#### 5.2.2 Descriptive statistical results

Two participants failed to complete the exposure condition manipulation due to an unknown computer error. Therefore, the data of the two participants was not included in any data analysis though they completed several measures following the exposure condition manipulation. The average age of the remaining participants was 19.88 (*SD* = 0.69), ranging from 18.25 to 21.42 years old. As in Study 1, the majority of participants were of Han nationality (176 Han and 2 Hui). Participants’ average BMI was 20.03 (*SD* = 2.72), with a range of 12.68 ~ 25.59. The mean values of body surveillance and body esteem across the three conditions were presented in Table 3.

**Table 3:** The mean values of body surveillance and body esteem across the three conditions in Study 2 (*M* ± *SD*).

	Fitness-appearance exposure condition	Fitness-performance exposure condition	Travel image exposure condition
Body surveillance	4.76 ± 1.14	4.00 ± 1.25	4.08 ± 1.32
Body esteem	45.83 ± 12.18	51.17 ± 13.00	52.42 ± 11.54

#### 5.2.3 Comparisons for body surveillance and body esteem across three conditions

**Figure 4:**
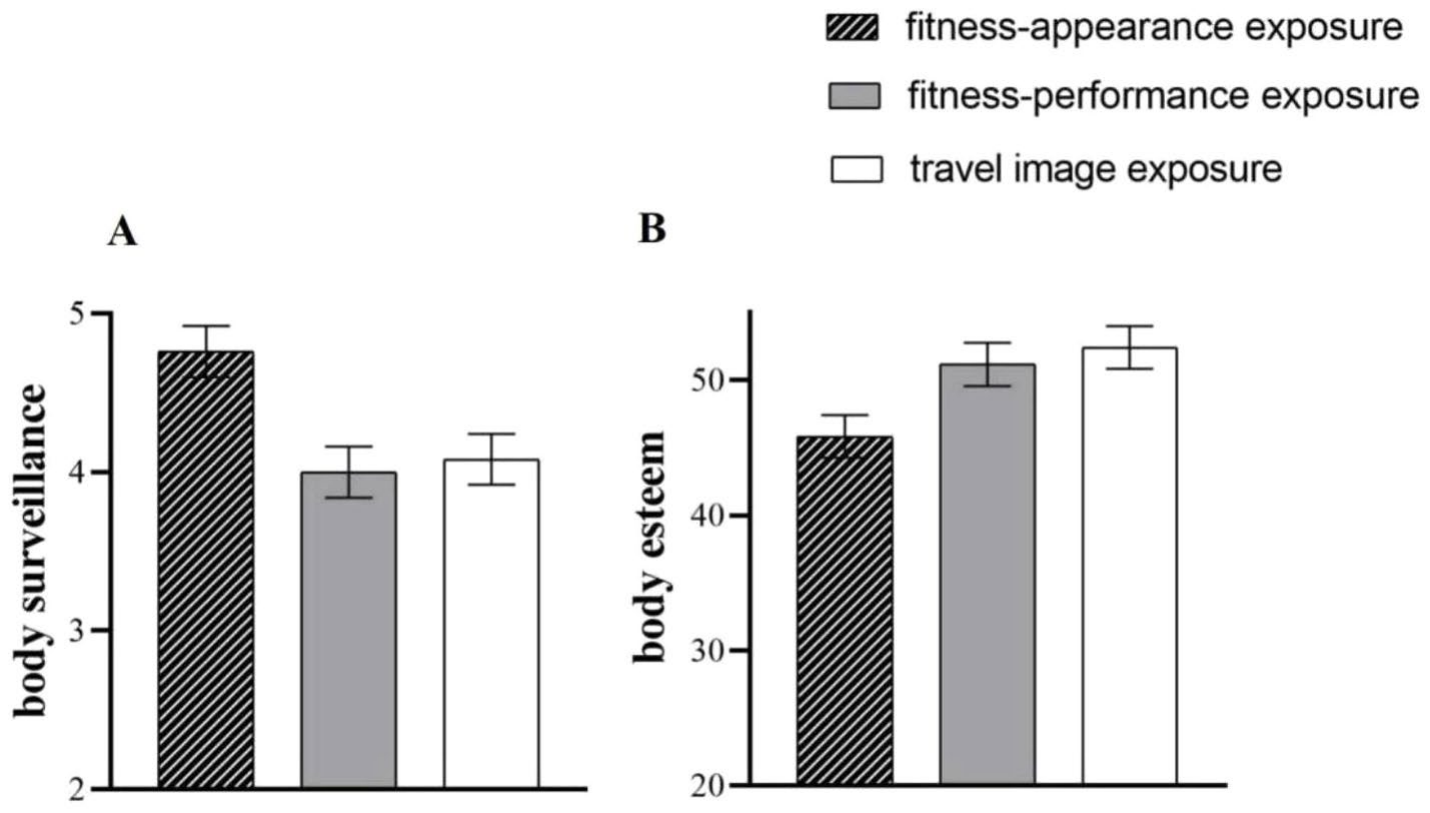
The comparisons for body surveillance **(A)** and body esteem **(B)** across the three conditions in Study 2 were presented schematically. Error bar means standard error.

A one-way ANOVA performed on body esteem revealed significant differences in body esteem among the three conditions, *F*(2,175) = 4.89, *p* = 0.009, partial *η*^2^ = 0.05. Planned comparisons showed that female body esteem in the fitness-appearance exposure condition was significantly lower than that in the travel image exposure condition, *t*(118) = −3.04, *p* = 0.003, *d* = 0.55, and also significantly lower than that in the fitness-performance exposure condition, *t*(116) = −2.31, *p* = 0.023, *d* = 0.43. However, there was no significant difference in female body esteem between the fitness-appearance exposure and travel image exposure conditions, *t*(116) = −0.55, *p* = 0.28, *d* = 0.11 (see Figure 4A). These findings provided direct evidence for Hypothesis 4 that appearance-relevant content involved in fitness posts, rather than pure fitness performance, harmed female body esteem. By demonstrating the causality between fitness posts exposure and female body esteem, Study 2 provided more convincing evidence for Hypothesis 1.

We also conducted a one-way ANOVA with body surveillance, which revealed a similar result pattern (in the opposite direction) to body esteem. Specifically, the ANOVA analysis showed that there were significant differences in body surveillance among the three conditions, *F*(2,175) = 6.76, *p* = 0.001, partial *η*^2^ = 0.08. Further analyses showed that body surveillance in the fitness-appearance exposure condition was significantly higher than that in the fitness-performance exposure and travel image exposure conditions, *t*(116)_appearance-performance_ = 3.45, *p* = 0.001, *d* = 0.64, *t*(118)_appearance-travel_ = 3.03, *p* = 0.003, *d* = 0.55, but there was no significant difference in body surveillance between the latter two conditions, *t*(116)_appearance-travel_ = −0.33, *p* = 0.75, *d* = 0.06 (see Figure 4B).

#### 5.2.4 The mediating role of body surveillance

To examine the mediating role of body surveillance between exposure condition and body esteem, we coded exposure condition as a dummy variable. As there was no significant difference in either body surveillance or body esteem between the fitness-performance exposure and travel image exposure conditions, such two conditions were both coded as 0, and the fitness-appearance exposure condition was coded as 1. Then, the Macro Process (model 4) by Hayes (2013) was applied to test the mediating role of body surveillance (bootstrapping 5,000 times). In line with Study 1, this analysis revealed a significantly indirect effect of body surveillance after controlling for the effects of global self-esteem, age, BMI, and subjective social status, *f* = −0.15, 95% CI [−0.28, −0.05]. The indirect effect of body surveillance accounted for 34.88% of the total effect. Specifically, exposure condition had a significantly positive prediction on body surveillance, *β* = 0.60, *p* < 0.001, and body surveillance had a significantly negative prediction on female body esteem, *β* = −0.26, *p* < 0.001. When the indirect effect of body surveillance was taken into account, exposure condition still had a significantly negative prediction on female body esteem, *β* = −0.28, *p* = 0.04. These results demonstrated the partial mediating role of body surveillance between exposure condition and female body esteem, thus providing additional evidence for Hypothesis 2.

### 5.3 Discussion

In Study 2, we randomly exposed participants to three kinds of image stimuli in the lab — fitness images containing appearance-relevant content, fitness images merely containing running performance, or travel images containing natural and cultural landscapes. Results showed that participants exposed to fitness images containing appearance-relevant content reported lower body esteem than participants exposed to travel images, while participants exposed to fitness images merely containing fitness performance did not report significantly different body esteem compared to participants exposed to travel images. These results indicated that appearance-relevant content involved in fitness posts, rather than pure fitness performance, contributed to harming female body esteem, thus providing direct evidence for Hypothesis 4.

As Study 1 in nature was correlational design, we could not draw causal inferences among variables. As a consequence, Study 1 indeed provided preliminary evidence for Hypotheses 1 and 2. In Study 2, we found that even temporarily exposed participants to fitness images containing appearance-relevant content in the lab circumstance, they still reported lower body esteem than participants exposed to travel images. Moreover, further analysis found that, consistent with Study 1, body surveillance played a mediating role between exposure condition and female body esteem. Thus, by clarifying the prediction direction among variables, Study 2 provided convincing evidence for Hypothesis 1 and 2. So far, the four hypotheses proposed in the present research have received complete support.

## 6 General discussion

In the current research, two studies were conducted to investigate the effect of fitness posts exposure on female body esteem and the underlying mechanisms. The results of Studies 1 and 2 consistently showed that fitness posts exposure produced a detrimental effect on female body esteem via the mediating role of body surveillance, thus supporting Hypotheses 1 and 2. In addition, Study 1 also found that appearance contingent self-worth moderated the mediating effect of body surveillance. That is, compared to participants with low appearance contingent self-worth, participants with high appearance contingent self-worth were more likely to suffer from the negative consequences of fitness posts exposure, thus supporting Hypothesis 3. In Study 2, we found that fitness posts containing appearance-relevant content, rather than fitness posts containing pure fitness performance, harmed female body esteem, thus supporting Hypothesis 4. The current research deepened our understanding of how exposure to fitness posts on social media harmed female body esteem.

### 6.1 Fitness posts exposure and body esteem

Social media use has been found to be related to a series of negative physical and mental consequences (Cataldo et al., 2021; Dobrean and Păsărelu, 2016; O’Day and Heimberg, 2021; Pop et al., 2022). However, in modern society, it is unrealistic for an individual to not engage in any social media activities throughout the day, because social media has become a part of our daily life (Siddiqui and Singh, 2016; Tartari, 2015). In this situation, it seems to be necessary for researchers to identify some specific activities that may raise potential detrimental effects on the mental health of users. For example, publishing and viewing selfies on social media platforms has been found to induce negative psychological consequences (Chang et al., 2019; McLean et al., 2019; Yao et al., 2020). Similarly, fitness posts exposure has been found to have close connections with negative body image, including negative body talk, body dissatisfaction, and low appearance self-esteem (Arroyo and Brunner, 2016; Prichard et al., 2018, 2020; Robinson et al., 2017; Tiggemann and Zaccardo, 2015). By first demonstrating the causality between fitness posts exposure and female body esteem, the present research provided new evidence for the close connections between fitness posts exposure and negative body image.

In Study 2, we further found that fitness posts containing appearance-relevant content, rather than fitness posts containing pure fitness performance, decreased female body esteem. Indeed, we are not the first to demonstrate that appearance-relevant factors intertwined with fitness will raise potential harm for female body esteem. For example, Prichard and Tiggemann (2008) have found that there was a significantly negative correlation between time spent exercising within the fitness center environment and female body esteem, while there was not a significant correlation between time spent exercising outside of the fitness center environment and female body esteem. They reasoned that the emergence of this phenomenon may be because the fitness center provided a platform where individuals made appearance comparisons with others (Prichard and Tiggemann, 2008). To our knowledge, this study is the first time to indicate that appearance-relevant factors involved in fitness activities, rather than pure fitness activities, may harm body esteem. Extending the research by Prichard and Tiggemann (2008), the present research indicates that not only in the offline context, a similar logic also exists in the online context. Namely, appearance-relevant content involved in fitness posts, but not pure fitness content, contributes to harming body esteem.

### 6.2 The mediating role of body surveillance

Consistent with Hypothesis 2, the present research found that fitness posts exposure decreased female body esteem partially via the mediating role of body surveillance. As we have reasoned, most fitness posts on social media platforms highlight appearance-relevant benefits (Carrotte et al., 2017; Jackson et al., 2021; Tiggemann and Zaccardo, 2016). So, it is reasonable that frequent exposure to such fitness posts increases body image concerns of women, resulting in higher body surveillance. However, due to the unattainableness of ideal bodies on social media (Yang et al., 2020; McComb and Mills, 2022), higher body surveillance will inevitably cause negative body self-evaluation of women, thus reporting lower body esteem.

It is worth mentioning that, although the exposure manipulation task was alleged as “a memory task” in Study 2, participants in the fitness-appearance condition still reported higher body surveillance than participants in the other two conditions. In other words, the state self-objectification of women was activated in an “unconscious and automatic” way. Indeed, the automatic activation of the state self-objectification among young females was also observed in prior research, including asking participants to try on and evaluate a swimsuit, exposure to media-portrayed idealized images, or merely presenting some words relevant to self-objectification (Gapinski et al., 2003; Monro and Huon, 2005; Roberts and Gettman, 2004). According to the propositions of the accessibility theory, the accessibility of specific knowledge constructs can be enhanced via a high use frequency of the knowledge constructs (Bargh et al., 1996; Higgins, 2012). Based on this, we attempted to speculate that, despite lacking solid evidence, fitness posts exposure on social media should be a common experience among young females, which enhances their susceptibility to objectifying cues and makes them frequently monitor their bodies without awareness in daily life. More importantly, frequent body surveillance can further cause various negative downstream consequences (e.g., lower body esteem).

### 6.3 The moderating role of appearance contingent self-worth

In Study 1, we found that appearance contingent self-worth moderated the mediation of body surveillance between fitness posts exposure and female body esteem. More specifically, for participants with high appearance contingent self-worth, fitness posts exposure significantly and positively predicted female body surveillance, while for those with low appearance contingent self-worth, fitness did not significantly predict female body surveillance. Appearance contingent self-worth reflects to what extent an individual determines the self-worth based on the appearance of the individual, which belongs to a specific domain of contingent self-worth (Crocker et al., 2003). As appearance often plays a dominant role in determining self-worth for women with high appearance contingent self-worth, we speculate that they will spend more time browsing fitness posts on social media than women with low appearance contingent self-worth. Consistent with this speculation, in Study 1, participants with high appearance contingent self-worth reported higher exposure frequency of fitness posts than participants with low appearance contingent self-worth. Given that appearance-relevant information is more accessible for women with high appearance contingent self-worth, we further speculate that, even exposure to the same fitness post, those women with high appearance contingent self-worth may be more likely to notice the objectifying cues involved in the post. Correspondingly, it is not surprising that they also more frequently engage in body surveillance (Modica, 2019; Moya-Garófano and Moya, 2019).

Upon failing to achieve the standard of ideal bodies on social media, women with high appearance contingent self-worth will display negative psychological and behavioral consequences (Moya-Garófano and Moya, 2019; Murray et al., 2016; Prieler et al., 2021). Due to the unreality of ideal bodies on social media, women often suffer from threats to self-esteem, especially to body esteem. Even in a given situation, a woman perceives that the self meets the ideal standard, the self-evaluation of the woman still will fluctuate with specific situations, which does more harm than good to the self-concept in the long term (Crocker, 2002). Compared to superficial aspects of the self (e.g., appearance), researchers have pointed out that individuals are more likely to function better when their self-evaluation is based on internal aspects of the self (e.g., competence; Crocker et al., 2004; Pyszczynski et al., 2003). Given that, educators in college can attempt to develop some prevention programs aiming at enhancing the self-worth of female undergraduates, whereby educators guide female undergraduates to accept themselves in an unconditional way or determine the self-worth based on competence rather than appearance. Off campus, for the sake of alleviating gender inequality, governments should encourage social media platforms to present kind reminders that the appearance of an individual is not equal to the worth of the individual when users browse fitness pots. According to our reasoning, these measures will help alleviate the self-objectification of women and elevate their self-evaluation.

### 6.4 Limitations and future work

Several limitations existed in the present research. Firstly, in Study 2, we found that there were significant differences in body surveillance between the fitness-appearance and fitness-performance conditions. According to our explanation, the appearance-relevant content should account for this difference between the two conditions. It should be pointed out that, participants in the fitness-performance condition were monotonously exposed to the screenshots of the running performances, while participants in the fitness-appearance condition were exposed to multiple kinds of exercises (e.g., running, yoga, and weightlifting). As prior literature has suggested that the relationship between fitness and female self-objectification varies with specific types of exercises (Prichard and Tiggemann, 2008), there was a slim possibility that specific types of exercises but not appearance-relevant content had accounted for the observed differences in body surveillance between the two conditions. Considering that we observed the same result pattern across two studies, we still have confidence in the reliability of our findings. In future research, we will further test this possibility.

Secondly, in both studies, body surveillance partially mediated the relationship between fitness posts exposure and female body esteem, which implies that there may be other mediating pathways between fitness posts exposure and female body esteem. According to the propositions of social comparison theory, individuals have an inner inclination to make comparisons with others to determine their self-worth (Festinger, 1954; Suls et al., 2002). According to the direction of social comparison, social comparison can be further divided into upward social comparison and downward social comparison. Compared to downward social comparison, upward social comparison often exerts more negative influences on the self-worth of individuals (Chang et al., 2019; Midgley et al., 2021). When women publish fitness posts, they often try their best to maximize physical attractiveness (e.g., displaying a slim figure or firm muscles). As a result, women will experience upward social comparison when accessing such fitness posts, which further harms their body esteem. In simple words, upward social comparison may mediate the relationship between fitness posts exposure and body esteem. This speculation should be investigated in future research.

Thirdly, in Study 1, we assessed the fitness posts exposure of participants at a general level and did not specify whether they were actively or passively exposed to fitness posts. In Study 2, participants were exposed to fitness images in a relatively passive way. Past research demonstrates that active and passive social media use motivations display completely opposite psychological consequences (Frison and Eggermont, 2017; Shaw et al., 2015; Valkenburg, 2022). As an example, Pan et al. (2023) found that passive TikTok use was negatively associated with female users’ body-esteem, whereas active TikTok use was positively associated with female body esteem. Given that, it may be necessary to conduct further work to investigate whether the effect of fitness posts exposure on female body esteem will vary with social media use type (active exposure vs. passive exposure).

Finally, only women were recruited as participants in both studies, which to some extent poses a threat to the generalization of our findings. Specifically, while a large body of research demonstrates that the self-objectification phenomenon is pervasive among women, some research reveals that the self-objectification phenomenon is also observed among men (e.g., Griffiths et al., 2018; Wang et al., 2024). That implies that, for some men (e.g., those with high appearance-contingent), fitness posts exposure may can increase their body surveillance, which further decreases their body esteem. Put differently, the result patterns observed in the present research may be suitable for a certain percentage of men. In addition to gender, there are other potential confounding factors that may affect the reliability or generalization of our findings. For example, we noticed that there were several participants with very low values in both studies. Such participants may be suffering from eating disorders, and they may perceive lower body esteem compared to those participants with normal BMI values. On a broad level, the present research was conducted in China-Mainland, whose sample consisted of female undergraduates. Thus, we cannot draw a definite conclusion about whether the existing result patterns can be extended to other age groups or culture backgrounds. From the perspective of research design, Study 1 in nature is a correlational design, and causal inferences among variables were tested in Study 2. Despite this, it is still possible that the relationships among variables are more complicated than we have supposed. For instance, due to the role of social media algorithms, those women with lower self-esteem may experience more fitness posts exposures (actively or passively), so that they can increase their physical attractiveness. However, according to our findings, exposure to online fitness posts will further decrease their body esteem. In this situation, fitness posts exposure and female body esteem actually have a reciprocal relationship. To thoroughly solve the above limitations, a large sample (including both men and women as participants) cross-cultural research may be needed in future, in which multiple research approaches should be employed to enhance the reliability and generalization of findings.

## 7 Conclusion

In the present research, two studies were conducted to examine how fitness posts exposure on social media affected female body esteem and the underlying mechanism. The results revealed that, the more frequently women were exposed to fitness posts, the more likely they perceived lower body esteem. Body surveillance played a partial mediation underlying this effect, but this mediation was further moderated by appearance contingent self-worth. Compared to women with low appearance contingent self-worth, those women with high appearance contingent self-worth were more likely to frequently monitor their bodies when exposed to fitness posts. The current research provides new insights into how fitness posts exposure has an influence on the self-evaluation of women.

## Data Availability

The raw data supporting the conclusions of this article will be made available by the authors, without undue reservation.

## Footnotes

^1^ http://www.afhayes.com
